# Digestive Enzyme Activity and Protein Degradation in Plasma of Heart Failure Patients

**DOI:** 10.1007/s12195-021-00693-w

**Published:** 2021-08-13

**Authors:** Vasiliki Courelli, Alla Ahmad, Majid Ghassemian, Chris Pruitt, Paul J. Mills, Geert W. Schmid-Schönbein

**Affiliations:** 1 Department of Bioengineering, Center for Autodigestion Innovation, University of California at San Diego, La Jolla, CA 92094-0412 USA; 2 Department of Chemistry/Biochemistry, University of California at San Diego, La Jolla, CA USA; 3 Department of Family Medicine and Public Health, University of California at San Diego, La Jolla, CA USA

**Keywords:** Trypsin, Lipase, Plasma peptide fragments, Intestine

## Abstract

**Introduction:**

Heart failure is associated with degradation of cell functions and extracellular matrix proteins, but the trigger mechanisms are uncertain. Our recent evidence shows that active digestive enzymes can leak out of the small intestine into the systemic circulation and cause cell dysfunctions and organ failure.

**Methods:**

Accordingly, we investigated in morning fasting plasma of heart failure (HF) patients the presence of pancreatic trypsin, a major enzyme responsible for digestion.

**Results:**

Western analysis shows that trypsin in plasma is significantly elevated in HF compared to matched controls and their concentrations correlate with the cardiac dysfunction biomarker BNP and inflammatory biomarkers CRP and TNF-α. The plasma trypsin levels in HF are accompanied by elevated pancreatic lipase concentrations. The trypsin has a significantly elevated activity as determined by substrate cleavage. Mass spectrometry shows that the number of plasma proteins in the HF patients is similar to controls while the number of peptides was increased about 20% in HF patients. The peptides are derived from extracellular and intracellular protein sources and exhibit cleavage sites by trypsin as well as other degrading proteases (data are available *via* ProteomeXchange with identifier PXD026332).

Connclusions

These results provide the first evidence that active digestive enzymes leak into the systemic circulation and may participate in myocardial cell dysfunctions and tissue destruction in HF patients.

**Conclusions:**

These results provide the first evidence that active digestive enzymes leak into the systemic circulation and may participate in myocardial cell dysfunctions and tissue destruction in HF patients.

**Supplementary Information:**

The online version contains supplementary material available at 10.1007/s12195-021-00693-w.

## Introduction

The fundamental mechanisms for damage to cardiac tissues during heart failure (HF) are currently uncertain.^14^,^30^ Whereas correlations with pancreatitis have been proposed, no consensus exists.^27^ We propose here a new hypothesis and provide initial evidence for a fundamental pathogenic mechanism that involves digestive enzymes.

After synthesis and discharge from the pancreas, digestive enzymes in the lumen of the small intestine are in high concentrations (~ mM), fully activated and relatively non-specific.^37^,^50^ They play the central role in daily degradation of large volumes of food constituents as the basis for nutrition. Autodigestion by one’s own digestive enzymes is prevented by compartmentalization of digestive enzymes in the small intestine provided by a barrier that consists of a layer of epithelial cells covered by mucin.^5^

However, digestive enzymes have also been found to leak across the mucin/epithelial barrier of the small intestine and participate in a number of pathophysiological processes, including severe multiorgan failure after different forms of shock.^2^,^6^,^7^,^25^,^31^ They cause a range of cell/organ dysfunctions including proteolytic degradation of plasma proteins, extracellular matrix proteins and membrane receptors, such as ectodomain cleavage of the insulin receptor, adrenergic receptors, and the vascular endothelial growth factor receptor 2, which causes insulin resistance, vasopressor resistance, endothelial apoptosis with capillary rarefaction, respectively,^8^,^41^,^48^ attenuation of the endothelial shear stress response,^3^ and other cell dysfunctions.^21^,^22^,^42^ In response to a high fat meal, digestive enzymes leak directly into the systemic circulation of even normal volunteers where they can be detected in an active form and capable of cleaving the insulin receptor.^26^

Patients with chronic HF have elevated intestinal permeability.^40^ We investigated in this study whether plasma of patients with American College of Cardiology/American Heart Association (ACC/AHA) Stages B and C HF contains selected digestive enzymes (trypsin, lipase), trypsin activity, and plasma peptides that could be generated by digestive proteases. We determined the digestive enzyme levels along with cardiac remodeling and inflammatory biomarkers that are elevated in HF, including brain natriuretic peptide (BNP), C-reactive protein (CRP), tumor necrosis factor-alpha (TNF-a), and interleukine-6 (IL-6).

HF is characterized by a desensitization and down-regulation of the β-adrenergic receptor (β-AR).^4^ We therefore examined β-AR sensitivity on peripheral blood mononuclear cells (PBMCs).^23^ The often-attributed mechanism of this phenomenon is elevated catecholamine levels, however, this does not fully account for the observation.^19^ In other cardiovascular diseases, including hypertension, unchecked protease activity in the peripheral circulation has been shown to cause proteolytic cleavage of the extracellular domain of the β-AR in arteries and arterioles.^22^

To carry out this investigation on HF patients, we rely here on blood samples collected at single timepoints to identify the presence and uncontrolled activity of selected digestive enzymes. The approach serves as a first step to identify digestive enzyme activity in the circulation of a group of patients only; activity measurements during a day in single individuals are outside the scope of the current study.

## Methods

### Study Participants

The sample consisted of 60 American College of Cardiology/American Heart Association (ACC/AHA) Stage B and C HF patients^13^ and 55 individuals without HF. The HF and control populations, respectively, compare as follows: age: 59.5 ± 11.4 yrs vs. 54.4 ± 10.7 yrs (*p* < 0.01); sex: 88% men vs. 64% (*p* < 0.001); body mass index: 30.2 ± 5.9 kg/m^2^ vs. 29.0 ± 10.9 kg/m^2^; currently a smoker: 14% vs. 16%. Participants were part of larger studies examining inflammation and mood in HF or cardiac rehabilitation.^24^,^52^

Patients were recruited from the cardiology clinics of the University of California, San Diego and the San Diego Veterans Affairs Medical Center. Non-HF individuals were recruited from the local community *via* advertisements and word of mouth referrals.

Inclusion criteria for all participants included age between 30 and 85 years, hypertension (> 140/90 to < 180/110 mmHg). Inclusion criteria for HF patients included symptoms of HF for at least 3 months which had been optimally treated with beta-blockers, diuretics and ACE inhibitors. In HF patients, left ventricular ejection fraction (LVEF) was assessed by echocardiography as part of routine medical evaluation. All human subjects research was carried out in accordance with the UC San Diego Human Research Protections Program and approved by one of the Program’s Institutional Review Boards. All participants provided written informed consent. The study was carried out in accordance with the Declaration of Helsinki principles.

Stage B and C HF patients and controls were recruited from sequential studies^24^,^34^,^35^ and grouped. In addition to Controls, Cohort 1 is comprised of Stages B & C patients and Cohort 2 has Stage C patients.

### Blood Sampling

Blood was drawn into EDTA-coated vacutainers (BD Biosciences, San Jose, CA, USA) *via* venous catheter while subjects had been fasting for the prior 12 hours. Samples were centrifuged at 4 °C for 15 min at 1700×*g* and the plasma stored at – 80 °C until analysis.

### Circulating Biomarker Assays

For the cardiac and inflammatory biomarkers, we selected a representative panel including BNP, CRP, TNF-a, and IL-6. Levels were determined following previously reported methods using enzyme-linked immunosorbent assays (ELISA) (Centaur BNP Assay, Bayer Diagnostics, New York, NY; R&D Systems, Minneapolis, MN; MSD Systems, Rockville, MD).^24^ Intra- and inter-assay coefficients of variation were < 5%. Neutrophil counts were obtained by flow cytometry.^36^

### β2-Adrenergic Receptor (β2-AR) Sensitivity Assay

For β2-AR sensitivity, as previously described,^34^ PBMCs were isolated from fresh blood and incubated with either 10 μM isoproterenol or phosphate buffered saline for 2 minutes. Following centrifugation, supernatants were then assayed for cyclic AMP levels (Perkin Elmer, Boston, MA). β2-AR sensitivity was expressed as the ratio of isoproterenol-stimulated cyclic AMP to non-stimulated cyclic AMP.

### Protein Quantification Using Western Blot

Total protein concentration in the plasma samples was determined using Pierce BCA Protein Assay (Thermo Fisher, Waltham, MA). General protein separation was carried out by electrophoresis on 4–20% gradient SDS-PAGE gels at 110 V for 1.25 h and transferred to nitrocellulose membranes (Bio-Rad Laboratories, Inc., Hercules, CA). Transfer membranes were then incubated in blocking buffer (1% BSA in TBST) at 4 °C under gentle agitation overnight. Blocked membranes were probed with primary recombinant monoclonal antibodies for trypsin (ab200997, 1:1000, Abcam, Cambridge, MA), pancreatic lipase (ab124915,1:30000, Abcam), and Transferrin (ab82411, 1:10,000, Abcam) before incubated in the corresponding secondary antibody (ab205718, 1/5000, Abcam). Membranes were incubated with Pico Chemiluminescent Substrate (Thermo Fisher Scientific) and then imaged *via* photo-sensitive autoradiography film (Genesee Scientific, San Diego, CA). Western blot images were digitally analyzed (gel analysis tool, Image J; NIH). The band density values for each of the western blots for trypsin and lipase were normalized to their respective transferrin values.

### Trypsin Activity Quantification Assay

Trypsin activity was quantified by use of a recent method.^26^ A trypsin-specific substrate (acetyl-N-DGDAGRAGAGK-C-NH_2_) (GenScript, Piscataway, NJ) was labeled with the Biodipy-FL-SE fluorophore (Invitrogen, Carlsbad, CA) by reacting equal volumes of the peptide substrate (10mg/mL) in 100 mM NaHCO_3_ (pH 8.2) with 10 mg/mL of the fluorophore in DMSO for one hour. Equal volumes of tagged substrate and plasma were allowed to react for 30 min at room temperature and then stopped by addition of EDTA. Aliquots of the mixture were loaded into polyacrylamide gels and then electrophoresed at 500V for 10 min in 0.5 × TBE running buffer. The gels were imaged using a BioDoc-It M-26 transilluminator (UVP, Upland, CA) at excitation and emission wavelengths of 302 and 500–580 nm, respectively. The fluorescent signal intensities were acquired by digital analysis (ImageJ).

### Peptide Extraction and Sequencing

#### High molecular weight protein depletion from human plasma

We combined 100 *µ*L of human plasma with 900 *µ*L of methanol. The samples were then mixed by vortex for 5 s at room temperature, kept for 30 min and then centrifuged at 12,000 rpm for 10 min, both at room temperature. 500 *µ*L of supernatant was transferred to a fresh tube. The samples were placed in speed-vac (to dry) to remove methanol from the samples. The samples were hydrolyzed in 0.5 mL of 0.5% formic acid and 5% acetonitrile (ACN) solution in preparation for C18 solid phase extraction. For solid phase extraction (WAT054955 Sep-Pak C18 1 cc Vac Cartridge, 50 mg, Waters Corporation, Milford MA) the manufacturer’s protocol was followed except for the elution step where a 40% ACN solution was used. The eluents were dried in speed-vac in preparation of mass spectrometry analysis.

#### Mass Spectrometry

A third of each sample was loaded for mass spectrometry analysis. Peptides were analyzed by ultra-high-pressure liquid chromatography (UPLC) coupled with tandem mass spectroscopy (LC–MS/MS) using nano-spray ionization. The nano-spray ionization experiments were performed using a Orbitrap fusion Lumos hybrid mass spectrometer (Thermo Fisher Scientific) interfaced with nano-scale reversed-phase UPLC (Dionex UltiMate™ 3000 RSLC nano System, Thermo Fisher Scientific) using a 25 cm, 75-micron ID glass capillary packed with 1.7-*µ*m C18 (130) BEH^TM^ beads (Waters Corporation). Peptides were eluted from the C18 column into the mass spectrometer using a linear gradient (5–80%) of ACN at a flow rate of 375 *μ*L/min for 2 h. The buffers used to create the ACN gradient were: buffer A (98% H_2_O, 2% ACN, 0.1% formic acid) and buffer B (100% ACN, 0.1% formic acid).

Mass spectrometer parameters were as follows; an MS1 survey scan using the orbitrap detector (mass range (m/z): 400–1500 (using quadrupole isolation), 60,000 resolution setting, spray voltage of 2400 V, Ion transfer tube temperature of 285 °C, AGC target of 400,000, and maximum injection time of 50 ms) was followed by data dependent scans (top speed for most intense ions, with charge state set to only include + 2–5 ions, and 5 s exclusion time, while selecting ions with minimal intensities of 50,000 at in which the collision event was carried out in a high energy collision cell (HCD Collision Energy of 30%), and the fragment masses where analyzed in the ion trap mass analyzer (with ion trap scan rate of turbo, first mass m/z was 100, AGC Target 5000 and maximum injection time of 35 ms).

Data analysis was carried out using the PEAKS Studio 8.5™ (Bioinformatics Solutions Inc., Waterloo, ON). PEAKS was also used to carry out label free quantification analysis with data filtering capabilities and statistical workflows such as cluster analysis.

### Quantitative In-Vitro Incubation of Plasma with Trypsin, Chymotrypsin, and Elastase

#### In-Vitro Addition of and Incubation with Trypsin, Chymotrypsin, and Elastase Followed by TMT Labeling and Depletion of Unlabeled Peptides with NHS-Activated Agarose Beads

50 *µ*L aliquots of Pooled Normal Human Plasma Na heparin (Innovative Research Inc., Novi, MI) for each condition was the starting material for each sample. Samples that served to test trypsin, chymotrypsin and elastase received 0.175 *µ*g of trypsin (Thermo Fisher Scientific, Waltham, MA), 0.0875 *µ*g of chymotrypsin (Promega, Madison, WI) and 0.0875 *µ*g of elastase (Promega), respectively. Ten conditions were created, including one no-enzyme sample with zero incubation time, one no-enzyme sample with overnight incubation, two samples with added trypsin and overnight incubation, one sample with chymotrypsin and overnight incubation, one sample with elastase and overnight incubation, two trypsin and chymotrypsin samples with overnight incubation, and two trypsin, chymotrypsin, and elastase samples with overnight incubation. All overnight incubation steps were at 37 °C for 20 h. The no incubation time samples were left at – 20 °C during the incubation period.

After overnight incubation, 5 *µ*L of 10% formic acid was added to each sample followed by desalting using a 30 mg Strata-XL 100 *µ*m polymeric reversed phase column (8B-S043-TAK, Phenomenex, Torrance CA). The eluent was dried in a speed vac and resuspended in 100mM triethylamonium bicarbonat (TEAB). The hydrated samples were labeled (TMT10; Thermo Fisher Scientific) for one hour and quenched using hydroxylamine. The samples were pooled and dried. After drying, the pooled sample was resuspended in 200 *µ*L of 8M urea in 200 mM ammonium bicarbonate and 4 *µ*L of 0.5 M tris(2-carboxyethyl)phosphine (TCEP; Thermo Fisher Scientific), 20 *µ*L of 400 mM chloroacetamide, and 600 *µ*L of 200 mM ammonium bicarbonate were digested with 0.5 *µ*g of trypsin overnight. Following the overnight digestion, the sample was desalted using a 10mg Strata-X 33 *µ*m polymeric reversed phase column (8B-S100-TAL, Phenomenex). The elution was dried and resuspended in 300 *µ*L of PBS, pH 8.5. Dried NHS-Activated Agarose Beads (5 mg, Pierce) was added and incubated at room temperature for 1 hour on a rotator. The solution was centrifuged at 1000×*g* and the supernatant was placed in a new tube, acidified, and desalted with the 100 *µ*L C18 tip (part#87783, Pierce). The final sample was dried and resuspended in 30 *µ*L 5% ACN, 5% formic acid (FA). 5 *µ*L were injected into the mass spectrometer for sequence analysis.

#### Mass Spectrometry

Analysis was carried out using ultra-high-pressure liquid chromatography (UPLC) coupled with tandem mass spectroscopy (LC–MS/MS) using nano-spray ionization. The nano-spray ionization experiments were performed using an Orbitrap fusion Lumos hybrid mass spectrometer (Thermo Fisher Scientific) interfaced with nano-scale reversed-phase UPLC (Thermo Dionex UltiMate™ 3000 RSLC nano System) using a 25 cm, 75-micron ID glass capillary packed with 1.7-*µ*m C18 (130) BEH^TM^ beads (Waters corporation). Peptides were eluted from the C18 column into the mass spectrometer using a linear gradient (5–80%) of ACN at a flowrate of 375 *μ*L/min for 120 min. The buffers used to create the ACN gradient were: buffer A (98% H_2_O, 2% ACN, 0.1% FA) and buffer B (100% ACN, 0.1% FA).

Mass spectrometer parameters are as follows; an MS1 survey scan using the orbitrap detector (mass range (m/z): 400–1500 (using quadrupole isolation), 60,000 resolution setting, spray voltage of 2200 V, Ion transfer tube temperature of 275 °C, AGC target of 400,000, and maximum injection time of 50 ms) was followed by data dependent scans (top speed for most intense ions, with charge state set to only include + 2–5 ions, and 5 s exclusion time, while selecting ions with minimal intensities of 50,000 at in which the collision event was carried out in the high energy collision cell (HCD Collision Energy of 38%) and the first quadrupole isolation window was set at 0.7 (m/z). The fragment masses were analyzed in the Orbi-trap mass analyzer with mass resolution setting of 15,000.

Protein identification and quantification was carried out using Peaks Studio 8.5 (Bioinformatics Solutions Inc.).

### Statistical Analysis

The experimental measurements (blood plasma trypsin and lipase and biomarkers BNP, CRP, and TNF-α, and β2-AR sensitivity) for the Control and the HF group are presented as Scatterplots along with mean and 95% confidence interval (CI). Difference in means between the Control and HF group were evaluated by Hotelling *T*^2^-test. 2-D Scatterplots were used to examine trends in plasma, to show comparison of means in 2-D scatterplots using the Hotelling *T*^2^ test for independent, heteroscedastic sample distributions. Means between groups were compared using *p*-values and CIs of the means from two-tailed, unpaired, heteroscedastic *t*-tests. The D’Agostino-Pearson omnibus normality test was used to determine if the data distributions were normal and to calculate the 95% CIs. Any data that was not normally distributed were log normalized prior to analysis. Group differences were determined with the Kolmogorov-Smirnoff (KS) test. Software tools were Matlab and GraphPad Prism (San Diego, CA).

## Results

### Pancreatic Trypsin, Lipase, Inflammatory Marker Levels and Neutrophil Count are elevated in HF Patient Plasma of Cohort 1 and Cohort 2

**Figure 1:**
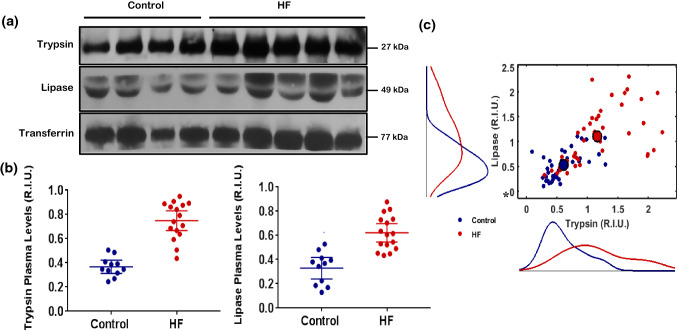
(a) Cohort 1. Representative western blots for trypsin (top row) and lipase (middle row) levels in blood plasma of Controls (lanes 1–4) and from HF patients (lanes 5–9). The bottom row shows Transferrin controls. (b) Cohort 2. Mean values and corresponding 95% CI of pancreatic trypsin and pancreatic lipase levels, detected *via* Westerns, in blood plasma of the Control (blue) and HF patient group (red). On average, (a) the trypsin level of the HF patient group was higher (*p* < 0.0001, *t*-test) and (b) the lipase level of the HF patient group was higher (*p* < 0.0001, *t*-test) compared to controls. The 2D-Scatterplot in panel C shows the variation of lipase vs. trypsin for the control (blue) and the HF (red) group along with the marginal distributions for lipase (left) and trypsin (bottom). Compared to the marginal distribution of the control group, the marginal distribution of the HF group was different for both trypsin (*p* < 0.0001, KS-test) and lipase (*p* < 0.0001, KS-test). The Scatterplot in panel c also indicates that, on average, compared to the control group, the elevation of trypsin in the HF group was accompanied by elevation of lipase, as the mean of the control group (large blue point) was statistically different from the mean of the HF group (large red point) (*p* < 0.0001, Hotelling *T*^2^-test).

**Figure 2:**
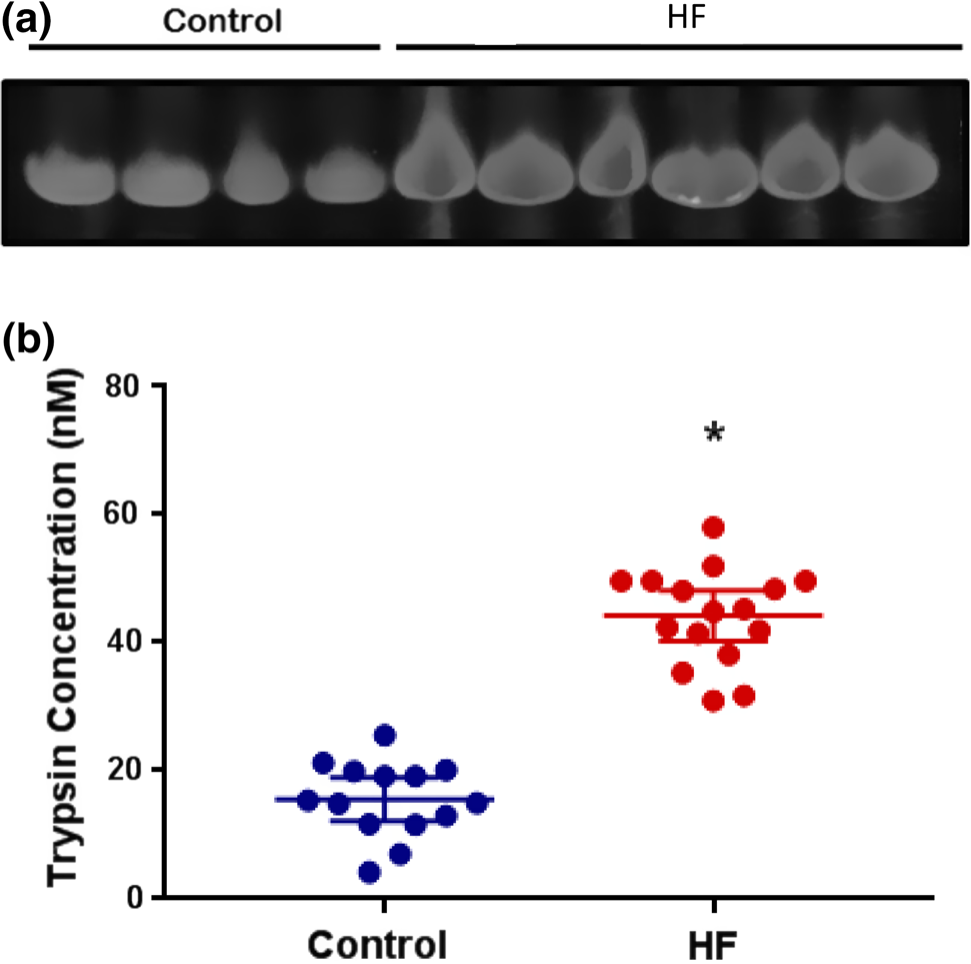
(a) Representative trypsin activity levels in blood plasma as detected by fluorescent substrate cleavage (Cohort 2); controls in lanes 1–4, HF patients in lanes 5–9. (b) Scatter diagram of equivalent trypsin concentration. The average equivalent trypsin concentration of the HF patient group (red) was significantly higher compared to the control group (blue) (*p* < 0.0001, *t*-test).

Western (trypsin and lipase) and ELISA analysis (BNP, CRP, TNF-a) showed significantly higher protein levels of both trypsin and lipase in the HF patients when compared to Controls in both Cohort 1 and Cohort 2 (*p* < 0.001 by t-test and KS test) (Figs. 1a and 1b; Supplement Fig. 1S A, B). 2D Scatterplot comparing trypsin to lipase demonstrated the presence of two different trypsin and lipase patient profiles in Cohort 1, where HF patients tended to have both higher trypsin and lipase values than Controls (Fig. 1c). This trend was validated using Hotelling *T*^2^ test analysis, which showed, on average, significantly higher trypsin and lipase means for HF patients compared to Control (*p* < 0.0001) (Fig. 1c). ELISA analysis of patient serum of Cohort 1 demonstrated significantly increased BNP (*p* = 1.9 × 10^−7^, Supplemental Fig. 2S A), TNF-a (*p* = 2 × 10^−4^, Fig. 2S B), and CRP (*p* = 2.47× 10^−7^, Fig. 2S C) in HF patients when compared to Control, but no significant difference was detected for IL-6 (*p* = 0.7262, Fig. 2S D). Neutrophil counts in whole blood were significantly higher in HF patients compared to Control (*p* = 2 × 10^−4^, Fig. 2S E).

### Β2-AR Sensitivity is Reduced in HF Patients

The isoproterenol stimulation response, an index of β2-AR sensitivity, was significantly attenuated in the HF group (*p* = 8.22 × 10^−6^, Fig. 2S F).

### Pancreatic Trypsin Activity is elevated in HF Patient Serum of Cohort 2

Blood plasma trypsin activity in Cohort 2, as detected by fluorescent substrate gel electrophoresis, was significantly higher in HF patients (*p* < 0.0001) compared to control (Fig. 2).

### Elevated Trypsin and Lipase Levels are Associated with Higher Inflammatory Marker Concentration

**Figure 3:**
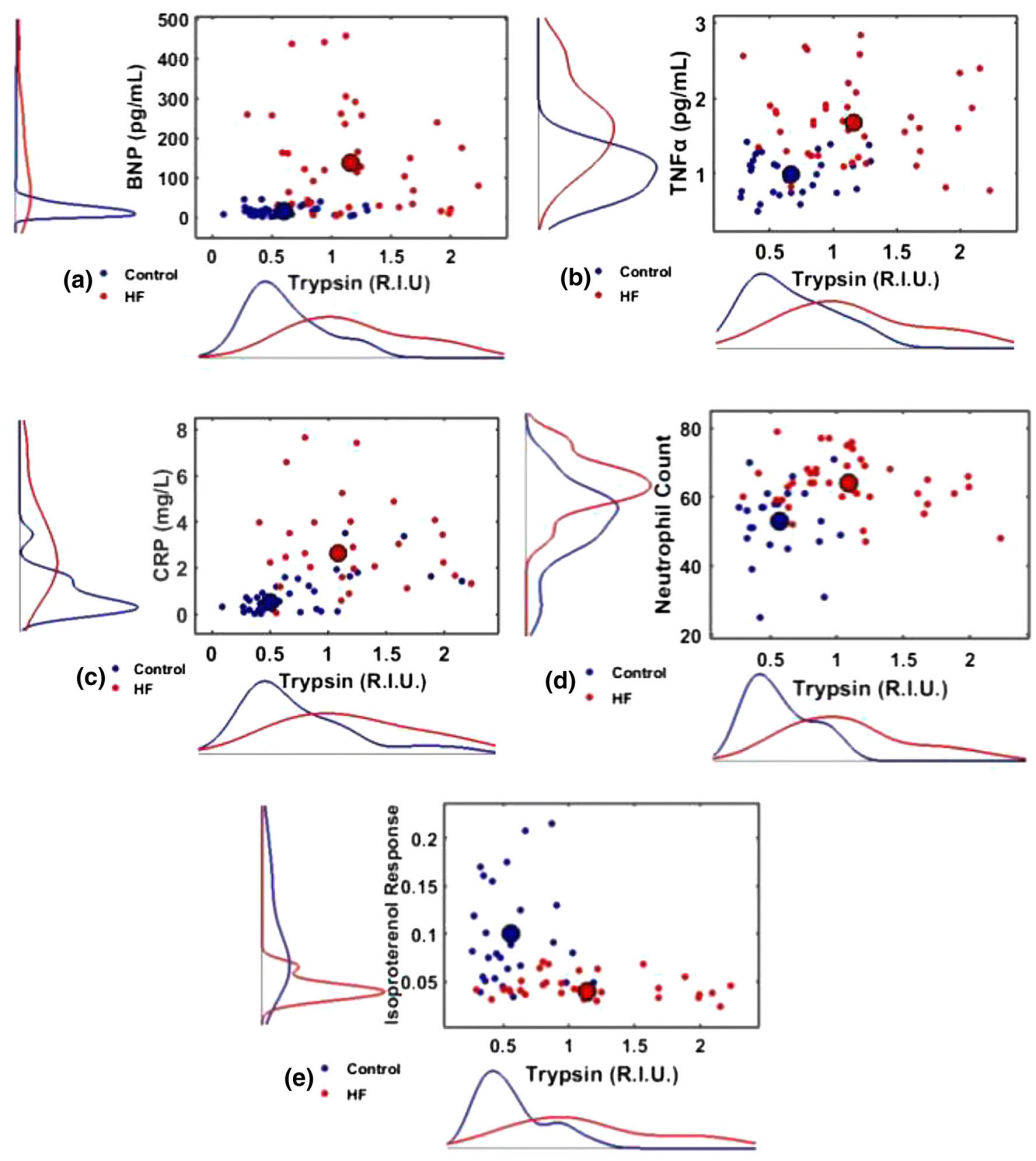
Cohort 1. 2D-scatter diagram of HF biomarkers vs. trypsin for Controls (blue) and HF (red) patients along with the marginal distributions for each biomarker (left) and trypsin (bottom): (a) BNP vs. trypsin, (b) TNF-α vs. trypsin, (c) CRP vs. trypsin, (d) Neutrophil Count vs. trypsin, and (e) Isoproterenol Response vs. trypsin. On average, compared to the Control group, the elevation of trypsin in the HF group was accompanied by (a) elevation of BNP, as the mean of the Control group (large blue dot) was statistically different from the mean of the HF group (large red dot) (*p* < 0.0001, Hotelling *T*^2^-test), (b) elevation of TNF-α (*p* < 0.0001, Hotelling *T*^2^-test), (c) elevation of CRP (Hotelling *T*^2^-test, *p* < 0.0001), (d) elevation of neutrophil count (*p* < 0.0001, Hotelling *T*^2^-test), and (e) depression of Isoproterenol Response (*p* < 0.0001, Hotelling *T*^2^-test). All Control marginal distributions were different from the corresponding HF distributions (*p* < 0.0001, KS-test).

**Figure 4:**
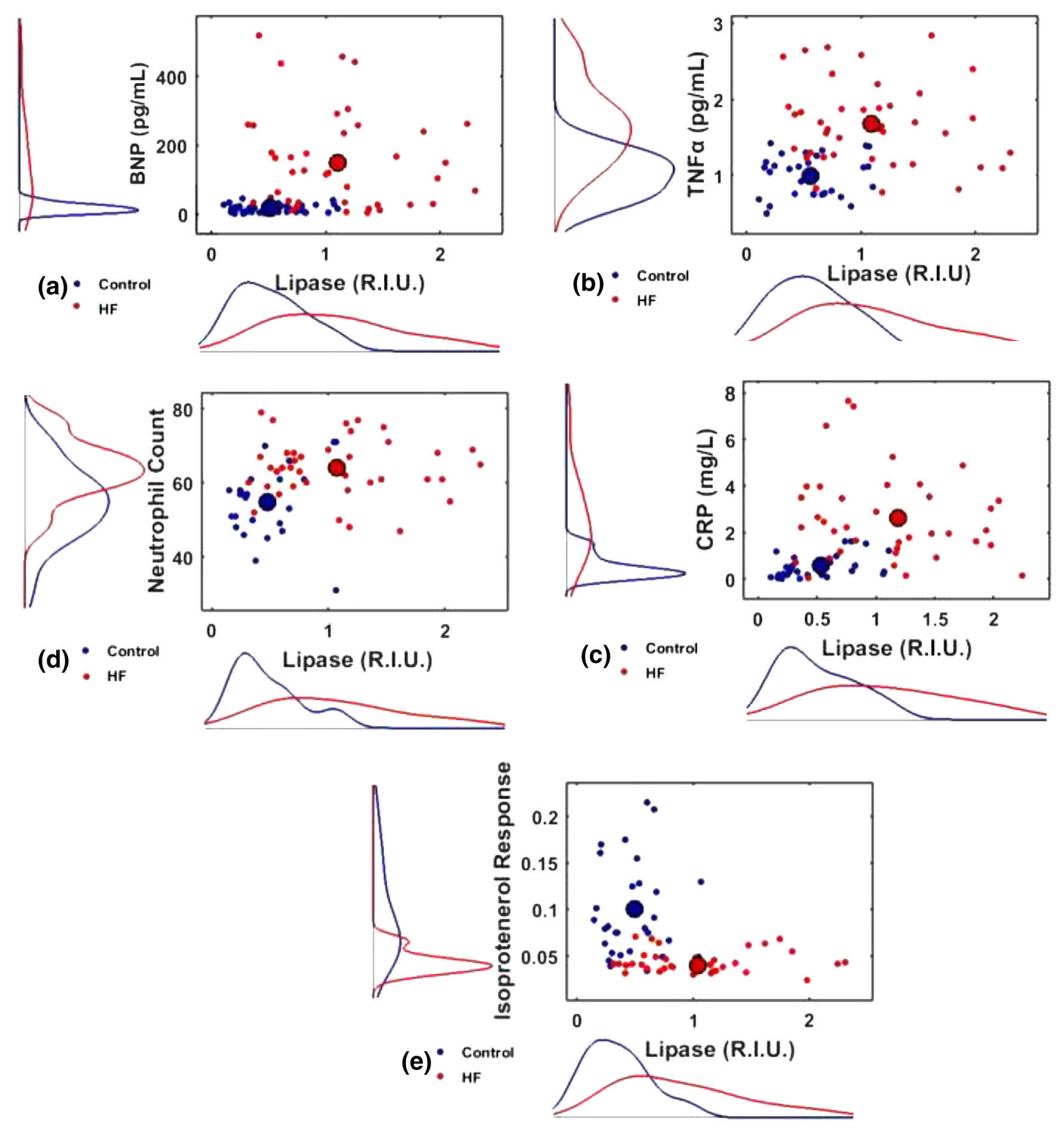
Cohort 1, 2D-scatter diagrams different HF biomarkers vs. lipase for the Controls (blue) and HF (red) patients along with the marginal distributions for: (a) BNP vs. lipase, (b) TNF-α, (c) CRP, (d) neutrophil count, and (e) Isoproterenol Response. Compared to the Control group, the average elevation of lipase in the HF group was accompanied by a significant elevation of (a) BNP (large blue and red dot represents mean of the Control and the HF group, respectively) (*p* < 0.0001, Hotelling *T*^2^-test), (b) TNF-α (*p* < 0.0001, Hotelling *T*^2^-test), (c) CRP (*p* < 0.0001, Hotelling *T*^2^-test), (d) neutrophil count (*p* < 0.0001, Hotelling *T*^2^-test), and (e) depression of Isoproterenol Response (*p* < 0.0001, Hotelling *T*^2^-test). All Control marginal distributions were different from the corresponding HF distributions (*p* < 0.0001, KS-test).

Since protein values of trypsin and lipase, inflammatory markers, and neutrophil counts were elevated in HF patients (Cohort 1), the correlation between these parameters were examined. On average, trypsin and inflammatory marker levels as well as lipase and inflammatory marker levels were significantly higher in the HF group (*p* < 0.0001 for all cases by Hotelling *T*^2^ test and KS test) (Figs. 3a–3c and 4a–4c). Similarly, when trypsin, lipase, and neutrophil count was analysed using a Hotelling *T*^2^ test, a significant average increase was noted in HF patients for both trypsin vs. neutrophil count (*p* < 0.0001, Fig. 3d) and lipase vs. neutrophil count (*p* < 0.0001, Fig. 4d).

### The Reduced β2-AR Sensitivity Response is Associated with Elevated Trypsin and Lipase Levels in HF Patients

The increased levels and activity of trypsin in HF patient plasma with potential proteolytic receptor cleavage by active proteases, were assessed using the isoproterenol response in neutrophils. Hotelling *T*^2^ test analysis demonstrated that for both trypsin and lipase, the means for trypsin or lipase vs. isoproterenol response were significantly lower in HF patients (Figs. 3e and 4e, *p* < 0.0001).

### Number of peptides and relative percentage of trypsin cleaved peptides are elevated in HF Patients

**Figure 5:**
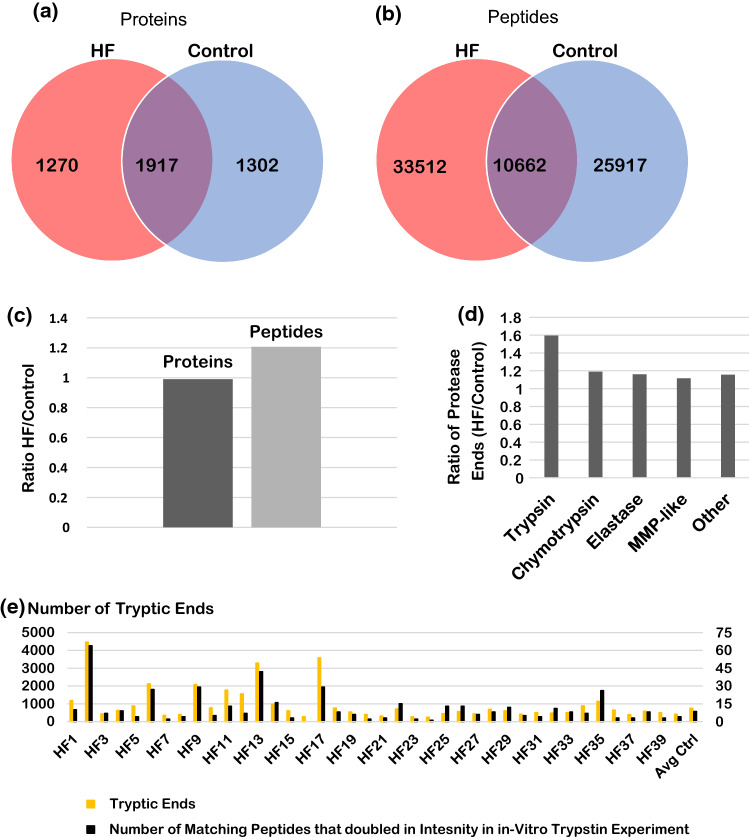
Stage C patients in Cohort 1, Venn diagrams comparing the identity of unique proteins (a) and the identity of unique peptides (b) identified by mass spectrometry in the combined datasets of all 40 HF patient samples (red) and all 40 control patient samples (blue). The bar graph (c) compares the total number of proteins and peptides between the combined datasets of all 40 HF patient samples to the combined data of all 40 control patient samples. As compared to the control, the aggregated HF patient data pool has a similar number of proteins identified, but about a 20% increase in the number of peptides identified by mass spectrometry. The bar graph (d) comparisons of the protease cleavage events are divided into 5 categories: Tryptic, Chymotryptic, Elastase, MMP-Like, and other. Cleavage is determined by the amino acid directly prior to the cleavage site in the peptides sequence. A trypsin cleavage must contain a K or R, chymotrypsin cleavage must contain a F, W, Y, L, or M, an elastase cleavage must contain a V, A, or G, a MMP-like cleavage must contain a S, A, G, N, E, and the other cleavage must contain a T, Q, P, I, H, D or C before the cleavage site. The bar graph (d) depicts the ratio of the combined HF patient sample dataset over the control aggregate dataset and shows that the combined HF dataset has about a 60% increase in Tryptic cleavage events, about a 20% increase for chymotrypsin cleavage events, about 15% increase in Elastase cleavage events, about 10% increase for MMP-like cleavage events, and about 15% for other cleavage events. The amount of tryptic cleavages is disproportionately increased as compared to the other types of cleavage. Taking a closer look at individual HF samples (e), the total number of trypsin protease cleavage sites (yellow bars, left vertical axis) for each HF patient sample are shown as bar graph with the addition of the average of the control samples at the right end of the graph (Avg Ctrl). The number of peptides of HF patients that overlap with peptides shown to at least double in intensity during an in-vitro experiment (by addition of trypsin to a commercial control human serum sample) are shown for each HF patient sample as black bars (right vertical axis). The peptides for the HF patients are listed in the Supplemental material (File: HF1 to HF40 Peptides of Figure 5).

To elucidate the degrading effects of an increased trypsin activity in plasma of HF patients, a mass spectrometric analysis of the endogenous free peptides in each patient samples (in Cohort 1) was carried out. Whereas the number of unique proteins in the aggregate HF data set (a total of 40 samples) and the aggregate Control data set was similar between the two groups, the HF sample pool had about a 20% increase in unique peptides identified compared to the Control (Figs. 5a–5c). Comparison of the protease cleavage events in these aggregate data sets (using the identity of the amino acid prior to cleavage site as the protease type indicator) illuminates any trends between the type of cleavage and the increase in the number of peptides identified. All protease cleavage events were increased in the HF sample pool as compared to the Control pool; however, the increase in the percent change of peptides cleaved by trypsin was disproportionately higher than that of other protease cleavages that were tested (Fig. 5d).

### Protein Groups in HF Patients Most Affected by the Increase in Trypsin Cleavages Events

To identify serum-based protease products that would result from increased trypsin activity, an *in vitro* experiment was carried out were these proteases were incubated with human serum and the abundance of cleavage peptide products where quantitatively monitored (see ‘Methods’). The peptides that showed more than twofold increase from a non-protease treated control where tabulated (ProteomeXchange Consortium, dataset identifier PXD026332)^32^ and compared to the peptides found in the HF patient samples.

The number of trypsin cleavage events seen by the presence of lysine or arginine prior to the cleavage location was mapped for each individual HF patient (in Cohort 1) and compared to the average of the Control patients (in Cohort 1) (Fig. 5e). These values are placed side by side with the number of peptides that intersect between that patient sample and a list of tryptic peptides that doubled in intensity after addition of trypsin in the in-vitro experiment (see ‘Methods’) (Fig. 5e). This analysis serves to elucidate that a specific subset of patients has abnormally elevated trypsin cleavage events that can be mapped to the presence of trypsin in normal human plasma.

This specific subset of patients was then used to identify protein groups that doubled in intensity in their plasma as compared to the average of the control (by means of NCBI’s DAVID software).^11^,^12^ The protein groups that had the largest number of proteins affected, and hence a higher enrichment score as determined by DAVID, were cold-shock proteins, cell-cell adhesion proteins, serine-protease inhibitors, alternate complement pathway proteins, SH3 domain containing proteins, direct complement pathway and coagulation proteins, extracellular matrix and collagen proteins, proteins that contain a homeobox, ribosomal proteins, proteins that contain a pleckstrin homology-like domain, and ATP binding proteins (Supplemental Table 1).

## Discussion

The current results in plasma of HF patients provide evidence for an enhanced level of digestive trypsin and pancreatic lipase. Whereas the elevated digestive enzyme levels correlate positively with inflammatory markers BNP, TNF-a, CRP and neutrophil counts, they exhibit an inverse correlation with the isoproterenol response that depends on β2-AR receptor function. The HF patient group has about an equal number of proteins compared to a control group, as detected by mass spectrometry, the HF patient group has an increased number of peptides in plasma derived from extra- and intracellular sources that may be of clinical relevance. The mass spectrometry peptide data in combination with an in-vitro experiment with purified digestive enzymes, provide further support for elevated digestive trypsin activity in plasma of HF patients.

When digestive enzymes in the small intestine leak across the mucin/epithelial barrier, for example after a high fat meal, several active degrading proteases are detectable in venous blood at levels up to 100 nM.^26^ In healthy individuals the activity is transient and returns to control values, after about two hours, but in individuals with preconditions, e.g. diabetics, the elevated plasma protease activity prevails over longer periods. These observations indicate that the full extent of protease activity is only visible if samples are investigated over hours, including postprandial periods. This brings to light a limitation of the current study, which was carried out on samples collected at one time point before breakfast. Future measurements of digestive enzyme activity should include meal schedules and contents as independent variables as well as gastrointestinal morbidities that may include mucosal barrier permeability to digestive enzymes.

Where is the source of the enhanced trypsin activity in the HF patients? There are two issues: (a) Do HF patients have significant sources of trypsin isoforms in tissues other than from the digestive tract, and (b) does pancreatic trypsin come from the pancreas and/or from the small intestine?

In regard to Issue (a): The monoclonal trypsin antibody (ab200997) in the current study reacts with pancreatic trypsin (trypsin-1) but can also bind to trypsin-3 and putative trypsin 6, but not trypsin-2 (according to the manufacturer, Abcam). Trypsin-3 has low protein levels in healthy (non-cancerous) tissue compared to the pancreas, duodenum and small intestine^16^ (*The Human Protein Atlas*). The expression may depend on prevailing pathology^39^ and it is likely that a small portion of trypsin-like activity has its origins in other tissues and blood leukocytes.^16^ The levels of different trypsin isoform, including trypsin 6, in cells and tissues of HF patients remain to be determined.

The gastrointestinal source of trypsin is further supported by the presence of other digestive enzymes. The monoclonal antibody to lipase (ab124915) is unique to the digestive tract. The elevation of both trypsin and lipase levels and their positive correlation is in line with a concurrent leakage of both digestive enzymes from the GI tract as compared to an increased exogenous trypsin production in other organs. Trypsin is smaller in molecular weight (~ 20 kDa for trypsin vs. ~ 50 kDa for lipase) so that its transport across the intestinal mucin/epithelial barrier is likely during lipase transport. This possibility is further supported by the presence of amylase (~55 kDa), as another digestive enzyme, in plasma of patients with heart failure and idiopathic dilated cardiomyopathy.^29^

Regarding Issue (b): Besides the pancreas, the high concentrations of digestive enzymes and their large volume in the small intestine constitute a major source of active trypsin. Increased intestinal permeability has been reported in inflammatory bowel disease, diabetes, and more recently HF, where leakage of pancreatic enzymes into the circulation is exacerbated by gut oedema and hypoperfusion.^40^,^45^,^49^,^51^ Whereas the rapid appearance of trypsin (within minutes) after a high fat diet meal^26^ suggests that trypsin acutely leaks out of the small intestine, under chronic HF conditions leakage out of the intestine and/or the pancreas are possible. Ultimately, to further resolve the question about the exact source of trypsin there is a need to trace its activity during food administration of isotopically labelled trypsin and its detection in serum using mass spectrometry.

The decreased isoproterenol activity and increased peptide fragments in HF patients suggests elevated levels of trypsin are capable of damaging cellular receptors and extracellular matrix components *via* proteolytic degradation. After establishing elevated plasma trypsin levels in HF patients, it became key to understand whether this trypsin was active and capable of causing tissue damage. Since myocardial tissue samples were not available in this study, the isoproterenol response in PBMCs served as a means of evaluating β2-AR functionality in active trypsin rich plasma. Decreased isoproterenol response in HF patients in the context of increased active trypsin suggests an active cleavage of β2-ARs by trypsin. In addition to the plasma membrane of PBMCs, β2-AR are also found on the myocardial surface as primary regulators of cardiac rhythm and cardiomyocyte depolarization. Therefore, significant damage of myocardial β-AR leading to a depressed receptor response, as seen in the PBMCs, would likely have a significant impact on cardiac functionality. HF has been previously associated with decreased myocardial β-AR, in part, due to receptor desensitization that has been attributed to the chronic catecholamine increase.^18^ However, myocardial β-AR damage has not been investigated as a mechanism for decreased receptor functionality^18^ although it was recorded in animal models.^38^ A role of trypsin-mediated myocardial tissue degradation was reported in dilated cardiomyopathy following influenza A infection and ischemic injury in myocardial infarction (MI).^20^,^28^ Ectopic trypsin production by cardiomyocytes following influenza A infection has been observed in murine models with myocardial inflammation and ventricular dilation.^28^ Treatment with aprotinin, a trypsin inhibitor, leads to a decrease in myocardial inflammation, MMP-9 activation, prevents ventricular dilation, and preserves myocardial function.^28^ The evidence is also in line with the cardioprotective effects with other serine protease inhibitors tested in the past.^33^ In humans, injury in myocardial ischemia and reperfusion, as measured by CRP and creatine kinase-MB levels, can be significantly inhibited with plasma-derived alpha-1 antitrypsin.^1^,^46^ In our patient cohorts, the presence of trypsin-mediated tissue damage is demonstrated by a significantly higher number of peptide fragments of extracellular matrix and cell junctional proteins in HF patients.

The trypsin in HF patients is not only associated with indications for tissue destruction but also markers of disease severity (e.g. BNP) and inflammation (e.g. TNF-a and CRP). While not explored in the past,^9^ the high values of active trypsin, BNP, and inflammatory markers suggest involvement of trypsin in HF disease severity and chronic inflammation. The correlation between neutrophil counts and trypsin also points to a role for trypsin in HF associated inflammation. Traditionally, elevated neutrophils are seen in acute inflammation, e.g. in the capillary no-reflow phenomenon,^10^ but recent evidence points to a role of neutrophils in chronic inflammation. In the case of HF, increased neutrophil count and lifespan has been reported, in addition to a significant inverse correlation between neutrophil count and left ventricular ejection fraction as well as neutrophil percentage and New York Heart Association stage.^47^ This behavior of neutrophils in HF has been hypothesized to be a response to inflammation in HF.^47^ Given the ability of trypsin to degrade all proteins and cause tissue destruction, as a documented trigger for an inflammatory response (which ultimately serves as a tissue repair mechanism;^43^), the current evidence points to the possibility that trypsin triggers and propagates formation of inflammatory markers in HF.

In addition to cleaving membrane receptors and plasma proteins, trypsin has the ability to activate proenzymes and prohormones as a relatively non-specific protease. In particular, it can activate pro-MMPs in HF^17^ and thereby amplify its degrading effect, including membrane receptor cleavage. Prothrombotic events in HF^44^ may be triggered by trypsin as an effective activator of prothrombin.^15^ Trypsin has been detected in acute animal models of myocarditis by influenza virus infection and proposed to activate proMMPs.^28^

The presented data are limited to plasma samples without analysis of myocardial tissue. Given the invasive nature of myocardial biopsy, it would not have been feasible to obtain tissue samples in the current study. Future studies of trypsin and other digestive proteases (including chymotrypsin, elastase) in HF need to determine (i) their contribution to either instigation or exacerbation of HF pathophysiology, (ii) protease inhibition as a treatment to slow or arrest myocardial function decline, (iii) digestive protease leakage from the intestine and mechanism for increased intestinal permeability, (iv) digestive protease plasma levels over time periods which include meals, and (v) the effects of trypsin on cells and extracellular matrix in the myocardium. Identification of active intestinal trypsin as a component of HF pathophysiology also provides a treatment target which could interfere with the trigger mechanism of tissue damage in the development of the disease.^53^

## Conclusion

The identification of increased pancreatic trypsin levels in HF patient plasma, which correlate with markers of active proteolysis and tissue damage, inflammation, as well as disease severity, points to the digestive protease trypsin’s involvement in HF pathology. Although further analysis is necessary to confirm its exact source, the circumstances in which trypsin and other digestive enzymes are released as a key instigator or propagator of myocardial tissue damage may open a new field of HF therapeutics which may preserve myocardial function.

## Supplementary Information

Below is the link to the electronic supplementary material.

Supplementary file 1 (DOCX 483 kb)

Supplementary file 2 (XLSX 16 kb)

## Abbreviations

HF: Heart failure
BNP: Brain natriuretic peptide
CRP: C-reactive protein
TNF-a: Tumor necrosis factor-alpha
IL-6: Interleukine-6
PBMC: Peripheral blood mononuclear cells
LVEF: Left ventricular ejection fraction
β2-AR: Beta2-adrenergic receptor
