# Supplementary material for: Digestive Enzyme Activity and Protein Degradation in Plasma of Heart Failure Patients
Source: Cell Mol Bioeng. 2021 Aug 13;14(6):583–96. doi: 10.1007/s12195-021-00693-w (PMC8630255; doi:10.1007/s12195-021-00693-w)

**Supplemental Material**

**Supplement Table 1**. Biological Function Clusters of Proteins with

Peptide Enrichment Score Greater Than 1*

***** Data are derived from six HF samples (HF2, HF6, HF9, HF12, HF13, HF17; see Figure 8B) with enhanced numbers of cleavage ends for trypsin/chymotrypsin compared to other HF samples.

| **Cluster Name** | **Average Enrichment Score** |
| --- | --- |
| ECM (structural constituent)/ collagen | 1.67 |
| Homeobox (DNA binding region) | 1.53 |
| Ribosomal | 1.45 |
| Pleckstrin Homology (-like domain) | 0.86 |
| SH3 Domain | 1.76 |
| ATP Binding | 0.30 |
| Cell-Cell Adhesion (junctions-cadherins) | 3.54 |
| Cold-Shock Protein | 3.95 |
| Serine-Protease Inhibitor | 1.82 |
| Direct Complement Pathway/Coagulation | 1.76 |
| Alternative Complement Pathway | 1.82 |

Figure 2A, 2B becomes Supplemental Figure 1


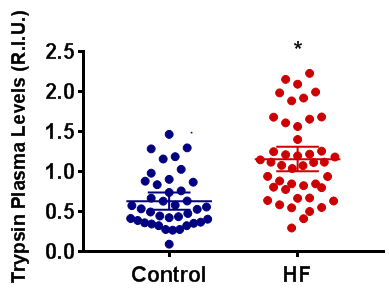

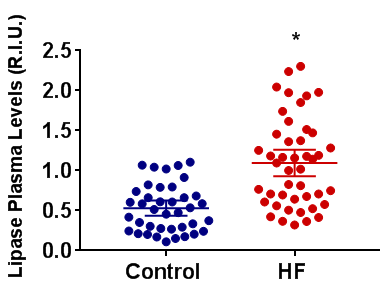

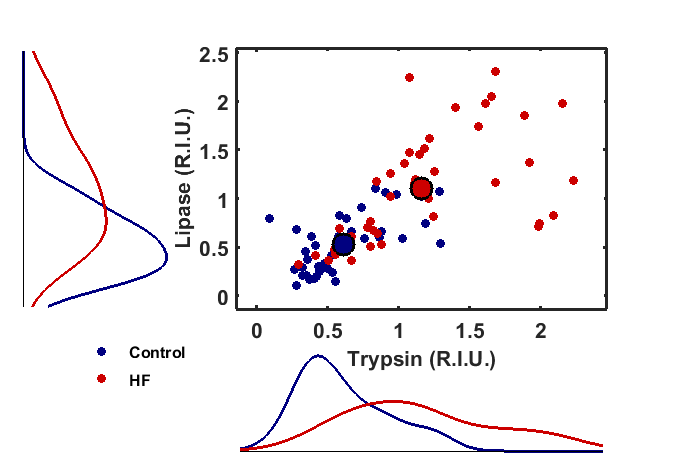


**C.**

Figure 3

**Figure 1S.** Cohort 1. Scatter diagram of pancreatic trypsin and pancreatic lipase levels in blood plasma of normal Control individuals (blue) and HF patients (red). 1D-Scatterplots show that, on average, compared to the Controls: (A) the trypsin level of the HF patient group was higher (t-test, p < 0.0001) and (B) the lipase level of the HF patient group was higher (t-test, p < 0.0001). In addition to data points, panels A and B show mean values along with corresponding 95% confidence interval. The 2D-Scatterplot in panel C shows the variation of lipase vs. trypsin for the Control (blue) and the HF (red) group along with the marginal distributions for lipase (left) and trypsin (bottom). Compared to the marginal distribution of the Control group, the marginal distribution of the HF group was different for both trypsin (p < 0.0001, KS-test) and lipase (p <0.0001, KS-test). The Scatterplot in panel C also indicates that, on average, compared to the Control group, the elevation of trypsin in the HF group was accompanied by elevation of lipase, as the mean of the Control group (large blue point) was statistically different from the mean of the HF group (large red point) (p < 0.0001, Hotelling t^2^-test).

Figure 3 becomes Supplemental Figure 2.

**Figure S2.** Cohort 1. 1D-scatter diagram (with mean values and corresponding 95% CI) of biomarker levels in the Control (blue) and the HF (red) groups. Compared to the Control group, the average (**A**) TNF-α level of HF patient group was higher (t-test, p = 2 × 10^-4^); **(B)** BNP level of the HF patient group was higher (p = 1.9 × 10^-7^ t-test); **(C)** CRP level of the HF patient group was higher (p = 2.47× 10^-7^, t-test); **(D)** IL-6 level of the HF patient group did not present statistically significant difference (t-test, p = 0.7262, t-test); **(E)** neutrophil count of the HF patient group was higher (p = 2 × 10^-4^, t-test); and **(F)** the isoproterenol ratio, an index of β-AR sensitivity, was lower in the HF patient group (p = 8.22× 10^-6^, t-test).


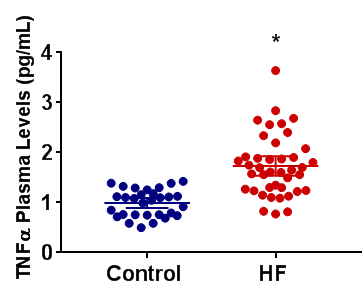

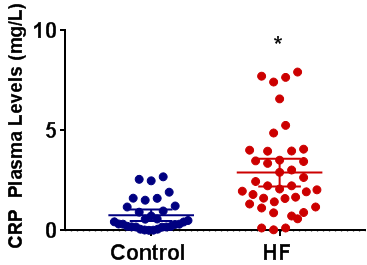


**C.**


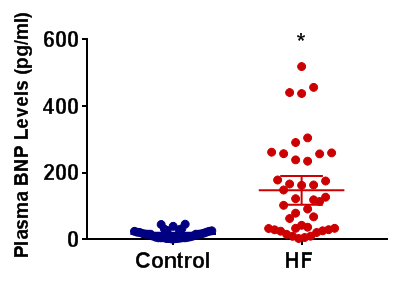


**A.**

**B.**


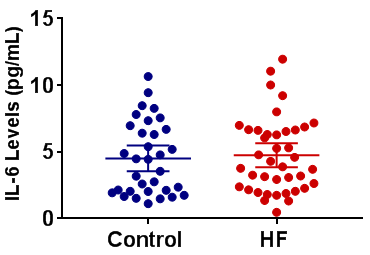


**D.**

**E.**


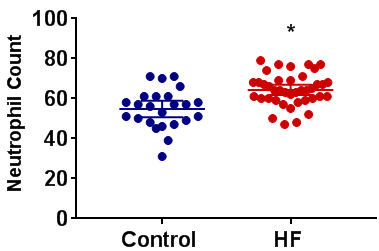


**F.**


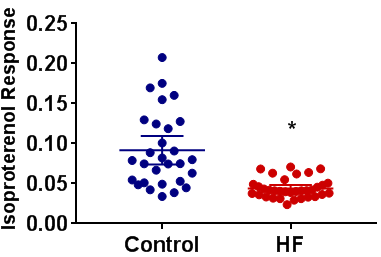

Supplement: Supplementary file 1 — (DOCX 483 kb) [file 12195_2021_693_MOESM1_ESM.docx]
